# Patient and physician perspectives of hand function in a cohort of rheumatoid arthritis patients: the impact of disease activity

**DOI:** 10.1186/s12891-016-1246-x

**Published:** 2016-09-15

**Authors:** Ana K. Romero-Guzmán, Víctor M. Menchaca-Tapia, Irazú Contreras-Yáñez, Virginia Pascual-Ramos

**Affiliations:** Immunology and Rheumatology Department, Instituto Nacional de Ciencias Médicas y Nutrición Salvador Zubirán, Avenida Vasco de Quiroga 15, Colonia Belisario Domínguez Sección XVI, Tlalpan, 14080 México City, DF México

**Keywords:** Musculoskeletal physiological processes, Rheumatoid arthritis, Disease activity

## Abstract

**Background:**

In 2004, we initiated an inception cohort of patients with recent-onset rheumatoid arthritis (RA). Hand function was incorporated into evaluations from 2014 onward. The objectives were to examine hand function in our cohort, compare hand function with function in healthy controls and determine the factors associated with impaired function.

**Methods:**

From February 2014 to June 2015, 139 patients (97.2 % of the cohort) had disease activity scored (28 joints, [DAS28]); the Michigan Hand Outcome Questionnaire (MHQ) and Disabilities of the Arm, Shoulder and Hand Outcome Measure (DASH) were completed, and the tip-, key- and palmar-pinch and grip strengths were measured. Sixty-nine healthy controls underwent the same evaluations. Ninety-nine patients underwent a second evaluation one year after their baseline. Descriptive statistics and linear regression models were used. Patients and controls signed informed consent.

**Results:**

Patients were primarily middle-aged females with a median disease duration of 7 years; 91 patients had DAS28-remission, and 16, 23, and 9 patients had low, moderate and high disease activity, respectively. Controls scored better than did patients with (any) disease activity level; remission patients had similar DASH and key pinch function as did controls with poorer MHQ and both tip and palmar pinch and grip strength. DAS28 was consistently associated with impaired hand function. Among the patients with a one-year re-assessment, changes in DAS28 correlated (rho = 0.34 to 0.63) with changes in hand function (*p* ≤ 0.01 for all comparisons), but there was no correlation with palmar pinch strength.

**Conclusions:**

Disease activity was associated with hand function impairment in RA patients with variable follow-up. MHQ discriminated poorer hand function in remission patients who otherwise had similar DASH scores as the controls did.

## Background

Rheumatoid arthritis (RA) is characterized by symmetric, polyarticular inflammation of the synovia, typically of the small joints of the hands, wrists and metatarsophalangeal joints of the feet [1]. Reports in the literature indicate that 70 % of all RA patients may present with some form of hand disability at follow-up [2, 3]. Eventually, such patients will be referred for prophylactic or reconstructive surgical interventions. However, there is limited evidence-based research investigating the factors that drive the surgical decisions for RA [4, 5]. Therefore, it appears convenient to identify patients with impaired hand function (HF) or those at risk for impaired HF early in the disease course and to identify potential predictors.

Although HF may be compromised at follow-up in the vast majority of RA patients, the current recommendations for disease assessment are limited to counts of swollen and tender joints, and these assessments do not include a comprehensive assessment of HF. Measurement of the grip strength using a dynamometer is a performance-based measure of HF that predicts long-term outcomes in RA patients [6, 7], and by using the appropriate equipment, grip strength may easily be incorporated into routine patient assessments.

Patient-reported outcomes are increasingly recognized as potentially being more accurate than physician-reported outcomes and laboratory parameters in predicting long-term disease consequences [8, 9]. Questionnaires included in routine evaluations examine how RA affects a patient’s physical function and ‘participation’ in daily activities, and the questionnaires include several questions pertaining to HF. However, these questions are usually limited to a few items [7, 10–13]. The Michigan Hand Outcome Questionnaire (MHQ) measures an individual’s perception of their hands in terms of the function, appearance, pain and satisfaction, all of which are reliable and valid measures of function in RA patients [14, 15]. The Disabilities of the Arm, Shoulder and Hand Outcome Measure (DASH) [16] questionnaire is a measure that was designed for use in single or multiple disorders of the upper limbs. DASH has demonstrated validity and reliability as a measure of physical disability in the upper extremities of RA patients [17, 18], and valid, reliable normative data are available for use in clinical and research settings [19].

Hand deformities that compromise HF are typical features in patients with longstanding and early RA onset [20, 21]. Impaired HF is prevalent in RA patients [22], and it correlates with clinical and laboratory parameters of disease activity [23, 24], patient disability [22] and hand deformity [24], although these correlations may vary according to the level of disease activity [25]. In addition, HF tests are sensitive tools for assessing the treatment response [26]. However, routine assessments of RA patients do not include HF evaluations. In 2004, we initiated an inception cohort of patients with recent-onset RA; the patients have been prospectively followed-up to date. In 2014, we performed a comprehensive evaluation of HF in our cohort of patients who were actively seen at the early arthritis clinic of our institution; these individuals had variable disease durations and disease activity. We hypothesized that a significant proportion of our patients could already present with (undetected) HF impairment, especially those with disease activity; we additionally aimed to identify the factors that are associated with impaired HF, with an emphasis on reversible factors.

The specific study objectives were as follows:

1. To describe HF in an inception cohort of RA patients who were classified according to their disease activity level (disease activity vs. remission) and to compare the HF of RA patients and healthy controls.
2. To determine the factors associated with impaired HF, with an emphasis on the disease activity as a potentially reversible factor.

## Methods

### Setting and study population

The early arthritis clinic of the *Instituto Nacional de Ciencias Médicas y Nutrición* (Mexico) was initiated in 2004. Candidates at the clinic had a disease duration of <1 year and no specific rheumatic diagnosis other than RA. Treatment was prescribed by the rheumatologist in charge of the clinic and was ‘treat to target’ oriented. Traditional disease-modifying anti-rheumatic drugs (DMARDs) were used in 99 % of the population, with or without corticosteroids (up to 50 % of the patients had corticosteroids during their follow-up). In 2014, when the study was approved and initiated, 143 patients were followed at the clinic (19 additional patients were lost to follow-up and two died) with variable disease duration, and all were invited to participate in the study. Through June 2015, 139 patients completed baseline assessments, and four were excluded for administrative reasons. In addition, 99 patients underwent a second evaluation that was performed one year after the baseline assessment.

### Standard clinical evaluations performed in the early arthritis clinic

When patients were enrolled in the clinic, their medical history and demographic data were recorded, as were their rheumatoid factor (RF) and antibody to cyclic citrullinated peptide (ACCP) levels. Follow-up evaluations were scheduled at regular intervals and, at minimum, always included swollen and tender joint counts, patient- and physician-reported outcomes, comorbidity and treatment assessments. Complete laboratory parameters were also determined at follow-ups as were X-rays of the hands and foot; the latter occurred on an annual basis [27].

### Evaluation of HF

HF was incorporated into the standard evaluations from 2014 onward. A brief interview preceded all testing and confirmed the absence of significant hand trauma; patients completed a validated Spanish version of the MHQ [14] and DASH [16]. Briefly, the MHQ contains 37 items that are distributed into six subscales that evaluate the overall HF as well as activities of daily living, pain, work performance, aesthetics and patient satisfaction with HF using a five-point ordinal scoring system. The MHQ takes approximately 15 min to complete. The scores range from 0 to 100; higher scores indicate better performance in all domains, except pain. Normative values are not available. DASH is a 30-item questionnaire with 21 physical function, six symptom and three social role-function items. A six-point ordinal scale grades the perceived difficulty for each task. The scale takes approximately 10 min to complete and 5 min to score, and the scores range from 0 to 100, where 0 represents the optimal HF.

**Fig. 1:**
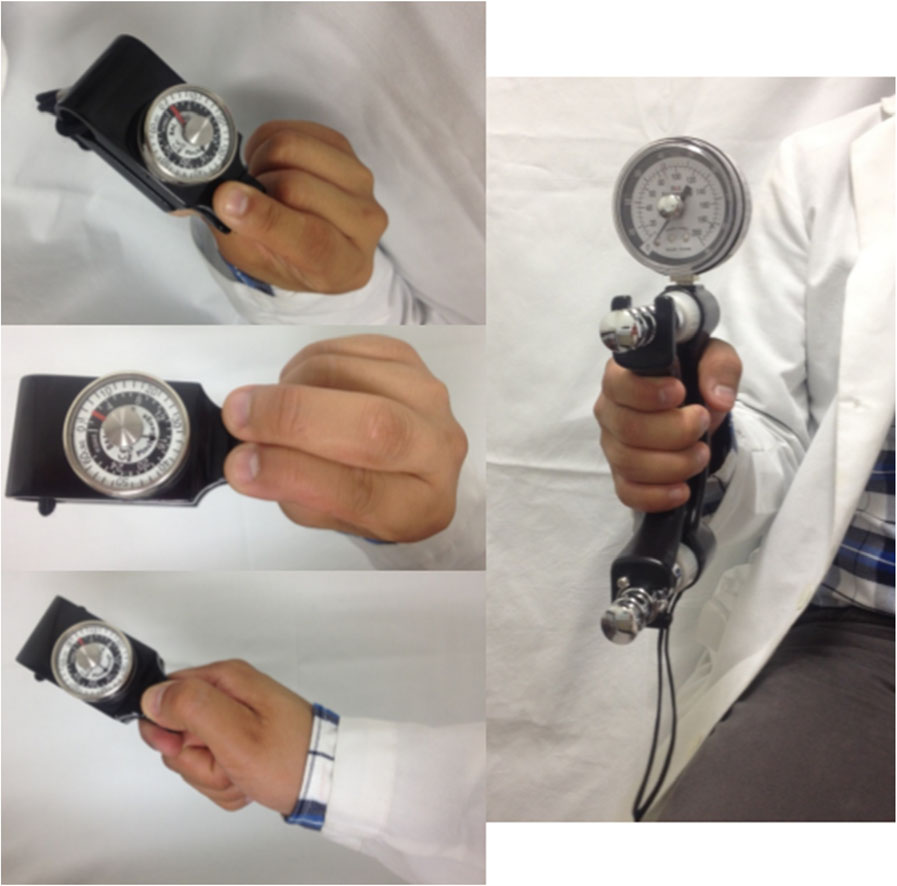
Position for grip strength and pinch measures

Subsequently, the tip pinch (two points), key pinch (lateral) and palmar pinch (three-jaw chuck) strengths were tested first, followed by grip strength. Standardized arm and hand positions were used as follows. Patients were seated in a comfortable position with their shoulder adducted and neutrally rotated, elbow flexed at 90°, forearm in neutral, and wrist between 0° and 30° dorsiflexion and between 0° and 15° ulnar deviation [28], (Fig. 1). A device (B&L Engineering®Hand Dynamometer, B&L Engineering, Santa Anna, CA, USA) was used to measure their grip strength. For standardization, the dynamometer was set at the second handle position for all patients. In addition, the B&L pinch gauge was used to measure the tip, key and palmar pinch strength, and scores were read on the needle side of the red readout marker. For each strength test, three successive measurements were recorded for each (dominant) hand, and the mean score was calculated. Both instruments were periodically calibrated during the study. All evaluations were performed by two previously trained assessors who were blinded to the rheumatic evaluations but not to the disease status.

### Control population

Sixty-nine healthy Mexican Mestizo adults lacking either a known medical condition (including disease of the upper limbs, limiting hand function and significant hand trauma) or treatment were included; these individuals comprised the control group. Control status was confirmed by interview and, when necessary, physical examination. Controls were matched according to age (±5 years) and gender because both affect grip strength. Controls were recruited from the hospital staff, students and patients’ relatives. MHQ and DASH questionnaires, tip pinch, key pinch, palmar pinch and grip strengths were measured as described for RA patients.

### Ethics

The study was approved by Internal Review Board of the *Instituto Nacional de Ciencias Médicas y Nutrición Salvador Zubirán,* Mexico. Patients and controls provided informed written consent to participate in the study.

### Statistical analysis

The distribution of the study variables was examined; data are presented as the (mean ± SD) for normally distributed variables and (median, Q_25_-Q_75_) for non-normally distributed variables.

Disease activity was defined according to DAS28 cut-offs [28]. Remission was defined with a more stringent criterion (DAS28 < 2.4), and analysis was repeated with remission defined as DAS28 < 2.6.

The MHQ score was converted into a dichotomous variable (within normal range/out of normal range) based on a cut-off obtained as the (mean-2SD) of control scores. The DASH score was converted to a dichotomous variable according to the following: within the normal range for a score ≤24.78 and outside the normal range for a score >24.78. This cut-off was established based on published normative data for the DASH questionnaire in a large general population survey [19]. Finally, data from local controls (mean + 2SD) were used to define values within the normal range for pinch and grip strengths. Pinch and grip strength analysis were separately performed for women and men. The analysis was repeated using normative data for adults [29], and similar results were obtained.

To achieve objective 1 (comparison of extended HF between controls, remission patients and patients with disease activity), multiple comparison post-hoc ANOVA with Tukey correction analysis was performed. Logistic regression models were used to establish the factors that were associated with HF impairment (objective 2). Variables were selected based on their clinical relevance and whether they emerged from the model as statistically significant (*p* ≤ 0.05) in the univariate analysis (*X*^2^, t Student’s and Mann-Whitney U tests were used as appropriate). Correlations between variables were also analyzed; to avoid overfitting the models, the variables included in the final models were based on the number of outcomes of interest. All statistical tests were two-sided and evaluated at the 0.05 significance level. Statistical analysis was performed using SPSS version 17.0 (IBM Corporation, USA).

## Results

### Characteristics of patients and controls

At study entry, RA patients (*n* = 139) were primarily middle-age (mean ± SD age, 44.1 ± 13.1 years) females (*n* = 124 [89.2 %]) with 11 ± 3.8 years of formal education, a median disease duration of seven years (interquartile range [Q_25_-Q_75_], 3 to 9 years) and inactive disease (median DAS28 of 2, [Q_25_-Q_75_] 1.3 to 3). One hundred twenty (86.3 %) patients had RF, 125 (90.6 %) had ACCP, 55 (39.6 %) had erosive disease and 80 (57.6 %) were prescribed corticosteroids. All patients were prescribed DMARDs; the median number of DMARDs/patient was 2 ([Q_25_-Q_75_] 1 to 2), and the median number of comorbidities/patient was 2 ([Q_25_-Q_75_] 1 to 3). Of the 69 controls who were evaluated, 62 (89.9 %) were female, and their mean age was 43.8 ± 11.5 years.

### HF evaluation in RA patients with disease activity, with remission and in controls

There were 91 patients in remission (median DAS28 of 1.5, [Q_25_-Q_75_] 1 to 2), 16 with low disease activity (DAS28 of 2.7, [Q_25_-Q_75_] 2.6 to 2.9), 23 with moderate disease activity (DAS28 of 4.4, [Q_25_-Q_75_] 3.7 to 4.7) and 9 with high disease activity (DAS28 of 6.3, [Q_25_-Q_75_] 5.8 to 6.8).

As expected, controls had significantly better scores than did patients with disease activity (low, moderate and high). However, RA patients in remission (DAS28 < 2.4) had similar DASH score and key pinch strength values as did the controls, but the RA patients in remission had worse MHQ scores and impaired grip, tip pinch and palmar pinch strengths (*p* < 0.005 for all comparisons) (Table 1).

**Table 1:** HF evaluation in controls, patients in remission and patients with disease activity

Variables^a^	Controls (*N* = 69)	Patients in remission (*N* = 91)	Patients with disease activity (*N* = 48)
MHQ	98.9 (95.5–100)	92.5 (83.3–97.7)*	65 (54.5–82.4)*
DASH	0 (0–1)	0.8 (0–6.7)	22.5 (5.4–41.5)*
Grip strength^b^	22.3 (19.1–26.5)	18.7 (13.7–23)*	10.7 (7.5–15.3)*
Tip pinch^b^	4.5 (3.9–5.4)	3.8 (3.2–4.5)*	3 (2.2–3.3)*
Key pinch^b^	7.1 (6–8)	6.3 (5.3–7.3)	4.8 (3.7–5.8)*
Palmar pinch^b^	6 (4.9–7)	5 (4–6.2)*	3.7 (2.5–4.6)*

*MHQ* Michigan hand outcome questionnaire, *DASH* Disabilities of the arm, shoulder and hand outcome measure

*p ≤ 0.005 vs. controls

^a^Data are presented as the median (Q_25_-Q_75_)

^b^Kg

Differences in the MHQ domains between RA patients in remission and controls were further explored. As shown in Table 2, there were significant differences between the groups in five of the six MHQ domains: overall HF, pain, work performance, aesthetics and satisfaction.

**Table 2:** Comparison of the MHQ domains between controls and RA patients in remission

	Overall hand Function^a^	Activities of Daily living^a^	Pain^a^	Work^a^	Aesthetics^a^	Satisfaction^a^
Controls	100 (98–100)	100 (100–100)	0 (0–0)	100 (100–100)	100 (93.8–100)	100 (94–100)
Patients in remission	95 (75–100)*	100 (100–100)	10 (0–30)*	100 (95–100)*	93.8 (75–100)*	91.7 (75–199)*

Data are presented as the median (Q_25_-Q_75_)

*MHQ* Michigan hand outcome questionnaire

*p ≤ 0.05

^a^MHQ domains

### Factors associated with impaired HF

#### According to MHQ

MHQ was considered to be within the normal range (MHQ-NR) for scores ≥84. Accordingly, 76 (54.7.3 %) patients had an MHQ-NR and 63 (45.3 %) did not. Patients in the former group had a lower DAS28 and fewer DMARDs/patient; they frequently tended to be male, younger and more educated and had a longer disease duration (Table 3). The logistic regression models that were used to identify the factors associated with MHQ-NR included the following variables: DAS28 and number of DMARDs/patient (low correlated with DAS28: rho = 0.36; *p* ≤ 0.001). DAS28 was the only factor associated with an MHQ score outside the normal range (β coefficient = 2.58 [95 % CI 1.79 to 3.73]; *p* ≤ 0.001; R^2^ = 0.358).

**Table 3:** Comparison of the demographic and disease characteristics between RA patients with/without HF within the normal range according to patient-reported outcomes (MHQ and DASH)

Characteristics	MHQ-NR, *N* = 76	MHQ below NR, *N* = 63	DASH-NR, *N* = 114	DASH above NR, *N* = 25	p1/p2
Female gender, N° (%)	65 (85.5)	59 (93.7)	99 (86.8)	25 (100)	0.17/0.07
Age at hand function evaluation^a^	42.7 ± 12.6	45.7 ± 13.6	42.5 ± 13.4	51.2 ± 8.8	0.18/0.002
Years of scholarship^a^	11.4 ± 3.6	10.5 ± 4	11.2 ± 3.7	10.1 ± 4.4	0.15/0.19
Disease duration, years^b^	7.5 (3–10)	6 (2–9)	7 (3–10)	4 (0–8)	0.17/0.007
N° (%) of patients with RF	63 (82.9)	57 (90.5)	97 (85.1)	23 (92)	0.22/0.53
N° (%) of patients with ACCP	69 (90.8)	56 (90.3)	102 (90.3)	23 (92)	1/1
DAS28^b^	1.5 (1–2.1)	3 (1.8–4.5)	1.7 (1.1–2.4)	4.4 (3–6)	0.000/0.00
N° (%) of patients with erosions	29 (38.2)	26 (41.3)	46 (40.4)	9 (36)	0.73/0.82
N° of comorbidities/patient^b^	2 (1–3)	1 (1–3)	2 (1–3)	2 (1–4)	0.55/0.56
N° (%) of patients with corticosteroids	41 (53.9)	39 (61.9)	64 (56.1)	16 (64)	0.39/0.51
N° of DMARDs/patient^b^	1 (1–2)	2 (2–2)	2 (1–2)	2 (2–2)	0.000/0.02

*RF* rheumatoid factor, *ACCP* antibodies to cyclic citrullinated peptides, *MHQ* Michigan Hand Outcome Questionnaire, *DASH* Disabilities of the Arm, Shoulder and Hand Outcome Measure, *NR* normal range, *p1* MHQ-NR score vs. MHQ score below NR, and *p2* DASH-NR score vs. DASH score above NR

^a^Mean ± SD

^b^Median (Q_25_-Q_75_)

#### According to DASH

One hundred fourteen (82 %) patients had a DASH-NR score >24.78, and 25 (18 %) did not. Patients from the former group were younger, had a longer disease duration, had a lower DAS28 and had fewer DMARDs/patient (Table 3). Logistic regression models that included the above-described variables showed that DAS28 (β coefficient = 4.08 [95 % CI 2.34 to 7.12]; *p* ≤ 0.001) and age (β coefficient = 1.08 [95 % CI 1.03 to 1.147]; *p* = 0.005) were associated with a DASH score out of the normal range (R^2^ = 0.612).

#### According to the pinch and grip strength

Impaired pinch and grip strengths were defined as follows, for women and men, respectively: tip pinch as <2.4 kg and <2.5 kg; palmar pinch as <3.4 kg and <2.9 kg; key pinch as <4.4 kg and <5.7 kg; and grip strength as <11.5 kg and <15.7 kg, respectively. The number of RA patients (women and men) with values outside the normal range for tip, palmar and key pinch and grip strengths were as follows: 26 (21 %) and 0; 34 (27.4 %) and 0; 32 (25.8 %) and 0; and 39 (31.5 %) and 2 (13.3 %), respectively.

A comparison between patients with/without impaired pinch and grip strengths was performed. Due to the limited number of men assessed, only data from the female population are presented. Variables with significant differences included (data not shown) DAS28 for tip, key and palmar pinch strengths and DAS28 with comorbidity/patient for grip strength.

Finally, different regression models were tested. Table 4 summarizes the most significant findings in the female subpopulation (*N* = 124). Higher DAS28 was consistently associated with impaired pinch and grip strengths; the only additional factor associated with impaired grip strength was the number of comorbidities/patient.

**Table 4:** Logistic regression models associated with impaired pinch and grip strength in the RA female subpopulation

	Tip pinch R^2^ = 0.255	Key pinch R^2^ = 0.341	Palmar pinch R^2^ = 0.288	Grip strength R^2^ = 0.394
DAS28	ß = 1.9 (1.4–2.5)^a^	ß = 2.2 (1.6–3)^a^	ß = 2 (1.5–2.7)^a^	ß = 2.3 (1.6–3.1)^a^
Comorbidity/patient				ß = 1.4 (1.1–1.8)^a^

*CI* Confidence interval; all *p* ≤ 0.001

^a^95%CI

**Fig. 2:**
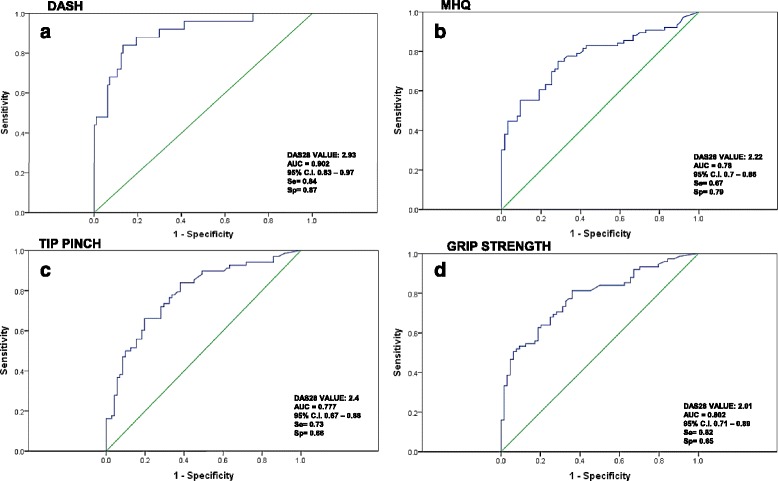
ROC curves: cut-off for DAS 28 to predict the MHQ-NR and DASH-NR scores, pinch and grip strength values within normal ranges

Receiver operating characteristic (ROC) curve analysis was performed to define the optimal cut-off for DAS28 to predict the MHQ-NR, DASH-NR, and pinch and grip strength values within NR (Fig. 2). DAS28 cut-offs varied from 2 to 2.9, depending on the selected outcome.

### Disease activity and impaired HF

**Fig. 3:**
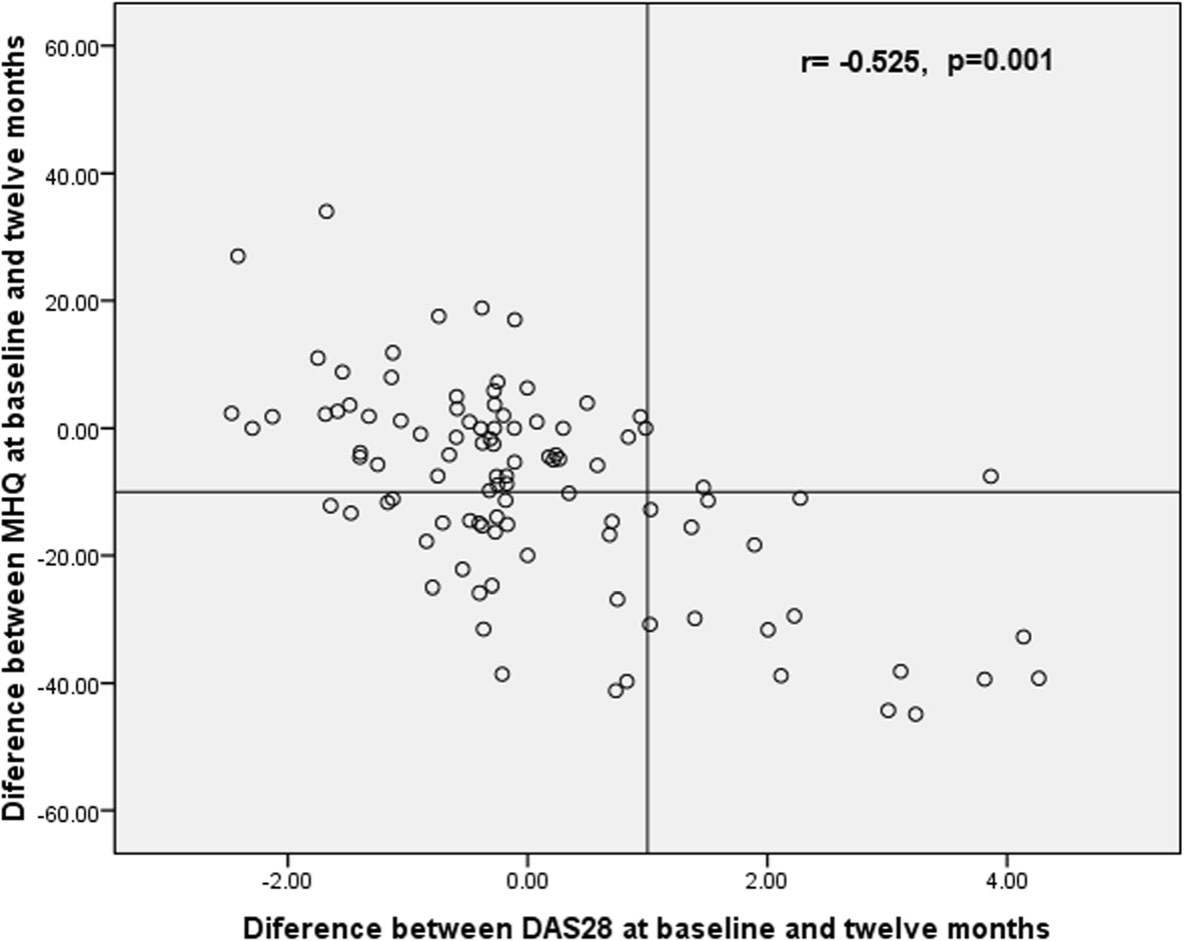
Correlation between changes in the DAS28 and MHQ (baseline to one year follow-up)

To further test the association between DAS28 and HF impairment, data from 99 patients who had an additional one year of follow-up assessments were analyzed. Of these, 53 (53.5 %) patients maintained the same disease activity level, 21 (21.2 %) improved and 25 (25.5 %) deteriorated. The changes in DAS28 correlated with the changes in MHQ (rho = −0.53), DASH (rho = 0.50), grip strength (rho = −0.55), tip pinch strength (rho = −0.34) and key pinch strength (rho = −0.63) (*p* ≤ 0.01 for all comparisons) (Fig. 3).

## Discussion

The present study was performed within an ongoing cohort of early-onset RA patients, who were highly represented by middle-age women with a median of seven years of disease duration and different disease activity levels. In addition to standardized rheumatic assessments, patients underwent an extensive HF evaluation that combined self-assessments and direct measurements of grip and pinch strengths. Similar combinations have been proposed as the most reliable evaluation of an individual’s functional capacity.

Disease activity evaluated according to DAS28 was the most consistent factor associated with impaired HF. The results were further confirmed in patients with prospective one-year assessments. Johnsson et al. [20] longitudinally assessed hand deformities in an early onset RA cohort and found that patients with deformities experienced more disease activity during the first five years. Although we did not evaluate hand deformities, they have been shown to affect both HF and general function [22, 23]. Additional studies have associated disease activity with the grip force, HF and functional abilities [25, 30, 31]. Interestingly, the DAS28 cut-offs for predicting HF impairment varied from 2.0 to 2.9, and they were generally below the DAS28 remission criterion that is most frequently used in clinical practice [32]. Our results support concerns raised against the DAS28 cut-off remission criterion, which overestimates true remission. DAS28 is a composite index that is widely used in clinical practice; however, patients in DAS28-remission do not necessarily perceive themselves as having HF within the normal range.

Age was the only additional (to DAS28) factor associated with HF impairment when it was evaluated according to DASH in the entire population. As described in the general population, older age is also associated (albeit inconsistently) with a more severe disease pattern and physical disability in RA patients [33, 34]. In our study, older age had a subtle impact on the DASH score. The number of comorbidity/patient was also associated with impaired grip strength, although the impact of DAS28 was stronger. These results were limited to the female population because there were few RA male patients with grip and pinch strengths within the normal range. There is strong evidence that the presence of comorbidities influences the outcome measures for the RA activity and severity; disease activity is usually assessed via composite indices, which include items influenced by concomitant disease [27, 35–37]. Additionally, in different populations, depression and other comorbid conditions are associated with worse patient-rated hand function [38, 39].

As expected, the present study showed that RA patients exhibited HF impairment compared with controls. Interestingly, RA patients in DAS28-remission had physical function (as per HAQ, data not shown) and HF similar to controls when evaluated according to DASH and key pinch strength. Our patients in remission did not achieve “control norms” when they were evaluated according to the MHQ. The MHQ is a patient-oriented questionnaire that covers HF problems, and it is particularly suitable for evaluating the rheumatoid hand [14]. We recommend that it be included in the RA patient evaluation because it may identify HF impairment in individuals who otherwise achieve population norms for health-related quality-of-life outcomes.

The limitations of the present study should be addressed. First, we performed an extended HF evaluation without including the complete International Classification of Functioning, Disability and Health (ICF) comprehensive core set for the measures developed for RA [40]. Second, we did not account for hand dominance when scoring the grip and pinch strengths, although studies have recommended ignoring this issue due to the small percentage (<10 %) of left-hand dominant subjects [29]. Third, HF assessments were performed by two differently trained physicians; we did not formally assess their agreement or reliability. Fourth, assessors were not blinded to the disease/control status and there could be bias in measures of grip and pinch strength. Fifth, we included a modest number of healthy controls from whom normative data were obtained. Sixth, we investigated a limited number of factors associated with HF impairment. Finally, our population only included a few men and our results may not be generalized to males.

## Conclusions

A comprehensive evaluation of HF in RA patients should be encouraged. Disease activity was the most consistent factor associated with impaired HF. We recommend including physical measures as well as patient reported-outcomes in the routine evaluation of HF in RA patients. RA patients who are in remission may present with HF that is similar to controls. In this regard, the MHQ preferentially identified HF impairment instead of patients in remission in whom the additional patient-reported measures were within the normal levels.

## Abbreviation

ACCP: Antibodies to cyclic citrullinated peptides
ANOVA: One-way analysis of variance
CI: Confidence interval
DAS28: Disease activity score for 28 evaluated joints
DASH: The disabilities of the arm, shoulder and hand outcome measure
DMARDs: Disease modifying anti-rheumatic drugs
HDA: High disease activity
HF: Hand function
ICF: International classification of functioning, disability and health
LDA: Low disease activity
MDA: Moderate disease activity
MHQ: The Michigan hand outcome questionnaire
NR: Normal range
RA: Rheumatoid arthritis
RF: Rheumatoid factor
SD: Standard deviation
